# Subscapularis Z-plasty With Coracoidectomy for Internal Rotation Contracture in Children With Brachial Plexus Birth Injury

**DOI:** 10.7759/cureus.47740

**Published:** 2023-10-26

**Authors:** Nizar Hamdi, Hend Alhamdan, Faisal Alshenaiber, Saleh Almutairi, Nouf Alturaiki

**Affiliations:** 1 Department of Orthopedic Surgery, King Faisal Specialist Hospital and Research Centre, Riyadh, SAU; 2 Department of Orthopedic Surgery, King Abdulaziz Medical City, Riyadh, SAU; 3 Department of Research, King Faisal Specialist Hospital and Research Centre, Riyadh, SAU

**Keywords:** bpbi, coracoidectomy, subscapularis lengthening, internal rotation contracture, brachial plexus birth injury

## Abstract

Objectives: Brachial plexus birth injury (BPBI) is a rare dystocia complication. Although it has a good prognosis, a significant number retain functional impairment to varying degrees. The data concerning shoulder function improvement and complication rates are conflicting due to variations in outcome measures between the studies. Therefore, we report our experience with this approach.

Methods: It was a retrospective study conducted at King Faisal Specialist Hospital and Research Center in Riyadh (FSH&RC), Saudi Arabia. Data such as patient demographics, Mallet scores, and passive external rotation (PER) in adduction and abduction were retrieved from the medical records.

Results: In active shoulder function, Mallet score significantly improved (P=0.00). The improvement was most remarkable in active external rotation movement (P=0.00) followed by hand to the neck. However, no significant gain was observed in active abduction and hand-to-back. At the final follow-up, with a mean of 2.9 years, the improvement in PER in adduction and abduction was maintained. Compared to six months postoperative, no significant difference was found in hand-to-neck, hand-to-back, and total Mallet score.

Conclusion: Subscapularis z-lengthening with coracoidectomy was consistently effective in correcting internal rotation contraction in a patient with BPBI. Significant improvements were observed in the Mallet score and PER in adduction and abduction.

## Introduction

Brachial plexus birth injury (BPBI) is a rare dystocia complication, affecting 0.04% to 0.3% of live births worldwide [1,2]. Although it has a good prognosis with a spontaneous recovery rate of 66% to 82%, a significant number retain functional impairment to varying degrees [3-5]. Internal rotation contracture of the glenohumeral joint is the most common disabling sequela of BPBI, attributable to weak abductors and external rotators and relatively hyperactive adductors and internal rotators. Historically, fixed internal rotation deformity has been correlated with glenohumeral dysplasia (GHD), which appears as early as six months old and progresses with age [6,7]. The shoulder contracture and function are unlikely to be corrected without surgical intervention.

The optimal timing, surgical approach, and specific indications for each type of surgery remain controversial. Although early intervention is advocated, the cut-off age is not well established. Nevertheless, the surgical intervention is delayed, probably due to a lack of an effective referral system and knowledge among the healthcare providers involved [8,9]. According to a recent recommendation by Muhlig et al., the operative treatment of shoulder internal rotation contracture is indicated when passive external rotation in adduction is less than 30° despite conservative management [10].

In 1934, tendon transfer to improve external rotation was first described by L’Episcopo et al. and has subsequently evolved, yet the principles remain the same [11,12]. Several authors reported improved shoulder function and GHD progression following latissimus dorsi and teres major transfer [13,14]. These results were promising. However, a recent long-term study showed significant deterioration in passive external rotation up to 23° over time [15].

Different subscapularis lengthening and release techniques were described because the subscapularis muscle has been linked to internal rotation contracture and GHD. We believe subscapularis Z-lengthening is superior to other release techniques, especially the subscapularis slide, which has a high recurrence rate unless combined with a tendon transfer [9,16]. Nevertheless, tendon lengthening, release, or transfer also risks losing midline function, which can be more functionally disabling. To our knowledge, only a few studies have been published about subscapularis Z-lengthening with coracoid process shortening/resection. This procedure was highly effective for releasing the shoulder contracture. However, the data concerning shoulder function improvement and complication rates are conflicting due to variations in outcome measures between the studies. Therefore, we report our experience with this approach.

## Materials and methods

It was a retrospective study conducted at King Faisal Specialist Hospital and Research Center (KFSH&RC), Riyadh, Saudi Arabia. The data were retrieved from the medical records. The study was conducted in accordance with the Declaration of Helsinki and was approved by the Institutional Review Board (Office of Research Affairs) at King Faisal Specialist Hospital and Research Centre, with an exemption from informed consent (approval number: 2231054).

Inclusion criteria were as follows: a child diagnosed with BPBI who underwent subscapularis lengthening with coracoidectomy at KFSH&RC, with a minimal follow-up of one year. In the hospital protocol, MRI under general anesthesia is used to delineate the glenohumeral joint for children aged five or below, while for those above five years, CT is used instead. The indication for surgery in this study was having internal rotation contracture despite physiotherapy. The exclusion criteria were as follows: a syndromic child, loss of follow-up, and underwent muscle transfers. Twenty-eight patients were enrolled in the study. Out of these patients, only five patients had primary nerve repair. No patient in this study received Botox injections.

Statistical analysis

Initially, a structured Microsoft Excel sheet (Microsoft Corporation, Washington, USA) was used to collect the data and then exported to SPSS Statistics version 25 (IBM Corp. Released 2017. IBM SPSS Statistics for Windows, Version 25.0. Armonk, NY: IBM Corp.) for analysis. The following were collected: patient demographics, including gender, side, age at surgery, and any complications. In addition, Mallet scores and passive external rotation (PER) in adduction and abduction were collected at three intervals: preoperative, at six months postoperative, and the last visit postoperative [17]. The improvement in global shoulder function was assessed using the Mallet score instead of the modified Mallet score because the operation does not affect midline function, and the reliability of the modification has not yet been published. The frequency and percentage (N, %) were presented for categorical variables, whereas the mean (standard deviation) for numerical variables. The means of numerical variables were compared using a paired t-test. Finally, a statistical test was considered significant if the p-value was less than 0.05.

Surgical technique

**Figure 1:**
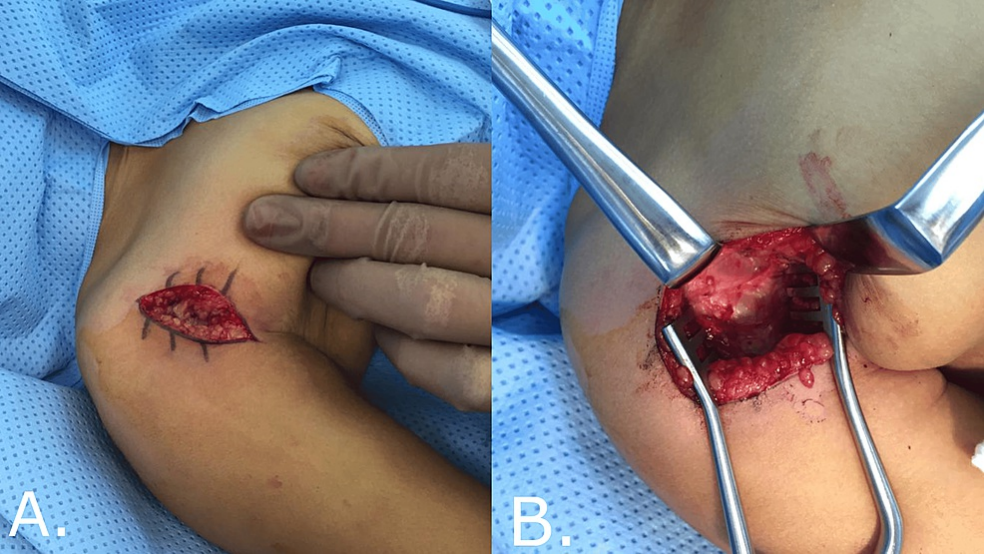
(A and B) Lazy S incision centered on the coracoid process utilizing the deltopectoral approach

**Figure 2:**
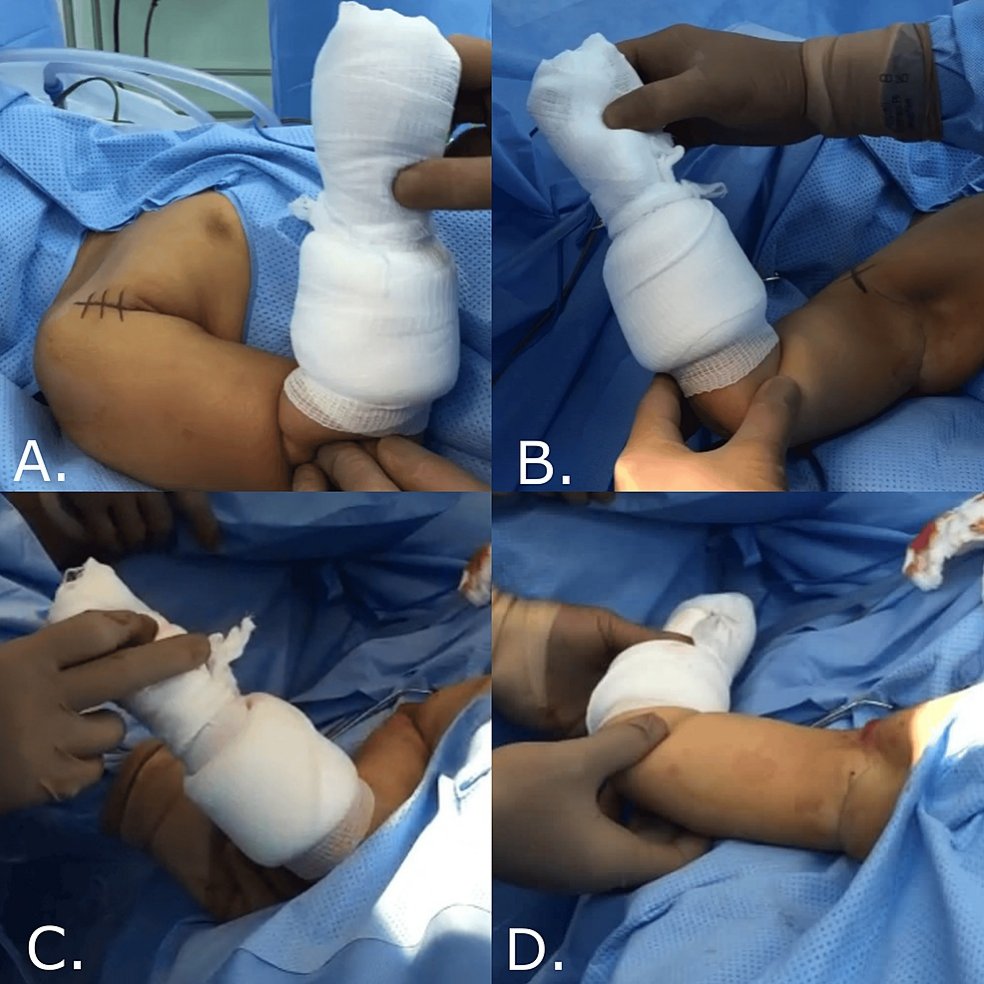
A four-year-old child with sequela of BPBI (upper lesion C5-C6). (A) Preoperative PER in adduction -10°. (B) Preoperative PER in abduction 40°. (C) Postoperative PER in adduction 45°. (D) Postoperative PER in abduction 90°

A senior consultant performed all procedures. After successful intubation, the patient was positioned supine. All the pressure points were protected. The patient was then prepped and draped in the usual sterile manner. We started using a deltopectoral approach longitudinal incision around 6-7 cm. The subcutaneous skin tissue was then dissected and the fascia opened (Figure 1). The cephalic vein was identified and mobilized. Then, the plane between the deltoid and pectoralis major developed. The conjoint tendon over the coracoid bone was identified next. Then, we subperiosteally cut the conjoint tendon and removed the coracoid bone's distal part. Then, we tested the PER in adduction to see if there was still severe internal rotation contracture, which was the case in the patients included in this study. Next, we proceeded to the subscapularis lengthening. The subscapularis muscle was identified and lengthened in a Z-plasty manner. It was done anteriorly from the insertion under the coracoid. We then did an open reduction of the glenohumeral joint by opening the capsule. Afterward, we sutured the subscapularis tendon in an external rotation position of about 45°. Then, the wound was closed in layers. At the end of the procedure, we applied a shoulder spica, keeping the shoulder at 90° and maximum external rotation (Figure 2).

## Results

Table 1 demonstrates the patients’ demographic data. A total of 28 patients were included; 22 (78.6%) were males, and the remaining six (21.4%) were females. The mean age of the included patients was 6.5. Most patients (69.7%) were affected by their right arm, compared to the left arm, which was affected in 13 patients (30.2%).

**Table 1:** Demographic characteristics Data are mean ± SD/N (%)

Variables		Mean ± SD/N (%)
Age at the surgical intervention (years)		6.5 ± 2.9
Gender	Male	22 (78.6)
	Female	6 (21.4)
Side	Right	12 (42.9%)
	Left	16 (57.1%)
Last follow-up (years)		2.9 ± 1.2

In active shoulder function (Table 2), the Mallet score significantly improved from 13.07 (1.6) to 16.25 (2.02) (P=0.00). The improvement was greatest in active external rotation movement (P=0.00), followed by hand-to-neck (+0.89, P=0.00) and hand-to-mouth (P=0.00). However, no significant gain was observed in active abduction and hand-to-back (P=0.184 and P=0.059, respectively).

**Table 2:** Mallet sub-scores and score at three intervals Data are mean ± SD, P≤0.05, * significant. N: number. A: paired t-test comparing preoperative with postoperative at six months. B: paired t-test comparing postoperative at six months with the last follow-up

Variable	N	Preoperative	Postoperative at six months	p-value ^A^	Last follow-up (2.9 ± 1.2 years)	p-value ^B^
Active abduction	28	3.64 ± 0.55	3.75 ± 0.41	0.184	3.71 ± 0.46	0.32
Active external rotation	28	2.07 ± 0.26	3.04 ± 0.83	0.00*	3.07 ± 0.97	0.83
Hand-to-mouth	28	2.75 ± 0.44	3.68 ± 0.54	0.00*	3.43 ± 0.97	0.050*
Hand-to-neck	28	2.46 ± 0.57	3.46 ± 0.63	0.00*	3.36 ± 0.78	0.37
Hand-to-back	28	2.32 ± 0.54	2.64 ± 0.78	0.059	2.68 ± 0.72	0.85
Total Mallet score	28	13.07 ± 1.69	16.54 ± 2.04	0.00*	16.25 ± 2.03	0.38

Table 3 presents the PER in adduction and abduction at three intervals. PER in adduction significantly improved from -14.4° (34°) preoperatively to 42.1° (30°.9) postoperatively (+56.5°, P=0.00).

**Table 3:** PER in ADD and ABD at three intervals Data are mean ± SD, * P≤0.05, significant. N: number, PER: passive external rotation, ADD: adduction, ABD: abduction. A: paired t-test comparing preoperative with postoperative at six months. B: paired t-test comparing postoperative at six months with the last follow-up

Variable	N	Preoperative	Postoperative at six months	p-value ^A^	Last follow-up (2.9 ± 1.2 years)	p-value ^B^
PER in ADD	28	-14.4 ± 34°	42.14 ± 30.9°	0.00*	51.1 ± 20.5°	0.09
PER in ABD	21	63.09 ± 20.02°	80.2 ± 26.6°	0.02*	83.75 ± 14.08°	0.48

At the final follow-up, with a mean of 2.9 (1.2) years, the improvement in PER in adduction and abduction was maintained. Compared to six months postoperative, no significant difference was found in hand-to-neck, hand-to-back, and total Mallet score, except for hand-to-mouth movement, which was statistically significant (-0.25, P=0.050) (Table 4).

**Table 4:** Overview of patient’s Mallet score before and after the surgery M: male, F: female, ABD: abduction, ER: external rotation, HM: hand-to-mouth, HN: hand-to-neck, HB: hand-to-back. a: the patient had primary nerve repair before. b: the patient developed internal rotation contracture recurrence

Patient number	Gender	Age (years)	Follow-up (years)	Preoperative						Postoperative (last follow-up)					
				ABD	ER	HM	HN	HB	Total Mallet	ABD	ER	HM	HN	HB	Total Mallet
1 ^a^	M	4	3	4	2	3	3	2	14	4	4	2	2	3	15
2	F	7	5	4	2	3	3	2	10	4	3	3	3	2	15
3	M	9	4	3	2	3	2	3	13	3	3	4	4	3	17
4	M	11	3	4	2	3	3	3	15	4	4	3	4	4	19
5 ^a^	M	3	5	4	2	3	2	2	13	3	4	4	4	3	18
6	M	5	3	3	2	3	2	2	12	3	2	4	4	2	15
7 ^a^	M	3	5	3	2	2	2	2	11	4	2	4	4	2	16
8	M	4	3	4	2	3	2	2	13	4	4	4	4	2	18
9	M	9	5	4	3	3	4	4	18	4	4	4	4	3	19
10	M	13	4	2	2	3	2	2	11	3	4	4	4	2	17
11	F	3	4	4	2	2	3	2	13	4	2	4	4	4	18
12 ^a^	M	5	3	4	2	2	2	2	12	3	4	2	2	2	13
13	M	8	4	4	2	3	3	2	14	4	2	4	4	2	16
14	F	3	3	4	3	3	3	2	14	4	2	3	3	3	15
15	M	6	3	3	2	2	2	2	11	3	4	2	2	2	13
16	F	6	3	4	2	3	3	3	15	4	4	4	4	3	19
17	M	6	2	3	2	3	2	2	12	3	4	4	4	3	18
18	M	11	1	4	2	2	2	3	13	4	2	3	3	3	15
19	M	4	3	3	2	2	2	2	11	3	4	3	3	3	16
20	M	6	2	4	2	3	2	2	13	4	2	4	3	2	15
21	M	6	2	3	2	2	2	2	11	4	4	4	4	3	19
22	M	7	2	3	2	3	2	3	13	4	4	4	4	2	18
23	M	4	3	4	2	3	2	2	13	4	2	4	4	2	16
24	M	8	2	4	2	3	3	3	15	4	2	4	3	2	15
25	M	14	1	4	2	3	3	3	15	4	4	4	3	4	19
26 ^b^	F	7	2	4	2	3	2	2	13	4	2	2	2	2	12
27 ^b^	F	6	2	4	2	3	3	2	14	4	2	3	3	3	15
28 ^a b^	M	5	1	4	2	3	3	2	14	4	2	2	2	4	14

Table 5 represents a simple linear regression. We investigated the relationship between the age at operation and the pre-post Mallet score outcome. The coefficient for the age preoperative was 0.14 (P=0.18) and 0.16 (P=0.21) postoperative, suggesting a positive but non-significant association between age and both the pre-post Mallet score outcomes.

**Table 5:** Linear regression model between the age at operation and Mallet Score Coefficient: the estimated coefficient representing the change in the dependent variable (pre- or postoperative) associated with a one-unit change in age

Variable	Coefficient		Standard error		p-value	
Age	Preoperative	Postoperative	Preoperative	Postoperative	Preoperative	Postoperative
	0.14	0.16	0.10	0.12	0.18	0.21

## Discussion

Despite BPBI's fair prognosis, 20-40% of cases retain permanent neurological deficits, even following primary nerve repair, leading to secondary structural shoulder deformities, most notably internal rotation contraction [18]. Clinical assessment of a child with a residual deficit in passive and active range of motion is critical for decision-making. In particular, the PER in adduction should be evaluated because the loss of this motion is associated with the progression of glenohumeral dysplasia [19]. In addition, the Mallet scale is widely used and validated for shoulder function assessment [18]. It assigns a score of 1 to 5 for active abduction, active external rotation, hand-to-neck, hand-to-back, and hand-to-mouth, and a total Mallet score is calculated from these scores, giving a maximum score of 25.

Since the early 20th century, secondary reconstructive surgeries have been used and modified to palliate the sequelae of BPBI. There has been reliable improvement in function following these surgical interventions, but full recovery cannot be achieved. Nevertheless, the impact of these procedures on the patients and their families should not be underestimated, as it affects their quality of life [20,21]. In 1971, Carlioz et al. attempted the release of the subscapular tendon from its origin, but the recurrence rate was high (50-70%) [22,23]. Later, isolated z-lengthening of the subscapular tendon with coracoid shortening was proposed [24]. A Z-plasty technique may reduce tension across the repair site and allow for greater postoperative motion. Moreover, the coracoid process of the scapula in BPBI is abnormally elongated and hooked inferiorly, which may complicate repositioning the subluxated humeral head. Few studies have been done on this technique compared to other procedures, such as tendon transfer, presumably because of reports of limited improvement and contracture recurrence rate [25].

Conversely, we report a significant gain in global shoulder function following subscapularis Z-lengthening. This study showed that the mean PER in adduction improved from -14.4° (34°) to 42.1° (30.9°) (+56.5°, P=0.00), and the mean Mallet score improved from 13.07 (1.69) to 16.25 (2.02) (+3.18, P=0.00). The study findings are comparable to those reported by Zayed et al., who showed a significant improvement in PER in adduction from -9° (11.4°) to 30.5° (11.9°) and Mallet score from 12.6 (1.09) to 17.7 (1.17) [26].

Among the Mallet sub-scores, active external rotation demonstrated the most significant improvement (+1, P=0.00), consistent with the findings of Van der Sluijs et al. and Kruit et al. [25,27]. We hypothesize that this improvement is attributable to the functional infraspinatus muscle, the primary external rotator of the shoulder. However, subscapularis muscle contracture, which is stronger and antagonizes the infraspinatus muscle, and impingement due to abnormal coracoid process preclude the proper preoperative evaluation of the infraspinatus muscle. Therefore, after subscapularis lengthening and coracoidectomy, the infraspinatus muscle resumes its preserved function. The surgery does not affect internal rotation; therefore, it is unsurprising that neither hand-to-back movements nor active abduction showed significant improvement after the procedure (P=0.184 and P=0.059, respectively).

The decline in shoulder function at mid- and long-term follow-up is one of the most undesirable outcomes, which has also been reported following other types of secondary reconstructive procedures for BPBI [28]. In this study, after a mean follow-up of 2.9 ± 1.2 years, the patients preserved their improvements in PER in adduction and adduction, abduction, active external rotation, hand-to-neck, and hand-to-back. However, a marginal yet statistically significant reduction was observed in hand-to-mouth movement (-0.25, P=0.050). The overall Mallet score remained stable throughout the follow-up period. Similar findings were reported by Gilbert et al. and Newman et al., who found that the gain in Mallet score and passive range of motion, but to a lesser degree, is maintained for an average of 3.5 to 6.5 years after the surgery [22,28]. On the other hand, a study by Kruit et al. found a significant decrease in the Mallet score after 6.5 years of follow-up [27]. This study was limited by the small number of patients (N=13) included in the analysis, and the Mallet sub-scores gain was maintained except for hand-to-back movement.

A well-documented complication of secondary reconstructive surgeries is deterioration in PER, causing recurrence of the internal rotation contracture [22,25,29]. While the exact cause has yet to be determined, it may be related to ongoing GHD or lifelong muscle growth abnormalities caused by muscle denervation at birth. In this study, three patients (10.7%) out of 28 had deterioration in PER (mean: from -16° to -36°), requiring a secondary external rotation osteotomy of the humerus at a mean follow-up of 1.6 years to place their hand in a functional position in front of their face. Van der Sluijs et al. identified severe BPBI (Naraks group 2 or 3) as the risk factor for this complication [25].

This study had some limitations that should be considered when interpreting the results. First, since this study is retrospective, the data quality and availability were reliant on the accuracy and completeness of the charts. For instance, this study did not measure some relevant variables, such as BPBI classification and shoulder CT preoperative, because they were missing. Second, the follow-up period was relatively short, but we will continue to follow these patients and report the results. Third, the sample size was small since a lesser portion of BPBI children developed an internal rotation contracture, which may have limited the power and precision of the analysis. Therefore, this study's results should be validated by larger and more robust prospective studies in the future.

## Conclusions

This study investigated the effectiveness of subscapularis Z-lengthening for treating internal rotation contracture in children with BPBI. The results showed that subscapularis Z-lengthening significantly improved the passive and active range of motion in the shoulder and global shoulder function, as measured by the Mallet score. The improvement was maintained at a mean follow-up of 2.9 years. However, three patients (10.7%) experienced deterioration in PER, which required a secondary internal rotation osteotomy.

The findings of this study suggest that subscapularis Z-lengthening is an effective treatment option for internal rotation contracture in children with BPBI. However, the risk of deterioration in PER should be considered. Larger and more robust prospective studies are needed to confirm the findings of this study.
